# Radiographic prediction of meningioma grade by semantic and radiomic features

**DOI:** 10.1371/journal.pone.0187908

**Published:** 2017-11-16

**Authors:** Thibaud P. Coroller, Wenya Linda Bi, Elizabeth Huynh, Malak Abedalthagafi, Ayal A. Aizer, Noah F. Greenwald, Chintan Parmar, Vivek Narayan, Winona W. Wu, Samuel Miranda de Moura, Saksham Gupta, Rameen Beroukhim, Patrick Y. Wen, Ossama Al-Mefty, Ian F. Dunn, Sandro Santagata, Brian M. Alexander, Raymond Y. Huang, Hugo J. W. L. Aerts

**Affiliations:** 1 Department of Radiation Oncology, Brigham and Women's Hospital, Dana Farber Cancer Institute, Harvard Medical School, Boston, Massachusetts, United States of America; 2 Department of Neurosurgery, Brigham and Women's Hospital, Harvard Medical School, Boston, Massachusetts, United States of America; 3 Department of Cancer Biology, Dana-Farber Cancer Institute, Boston, Massachusetts, United States of America; 4 Department of Pathology Brigham and Women's Hospital, Harvard Medical School, Boston, Massachusetts, United States of America; 5 The Saudi Human Genome Project, King Abdulaziz City for Science and Technology and Research Center at King Fahad Medical City, Riyadh, Saudia Arabia; 6 Center for Neuro-Oncology, Dana-Farber Cancer Institute, Boston, Massachusetts, United States of America; 7 Department of Radiology, Brigham and Women's Hospital, Harvard Medical School, Boston, Massachusetts, United States of America; Centre Hospitalier Sainte Anne, FRANCE

## Abstract

**Objectives:**

The clinical management of meningioma is guided by tumor grade and biological behavior. Currently, the assessment of tumor grade follows surgical resection and histopathologic review. Reliable techniques for pre-operative determination of tumor grade may enhance clinical decision-making.

**Methods:**

A total of 175 meningioma patients (103 low-grade and 72 high-grade) with pre-operative contrast-enhanced T1-MRI were included. Fifteen radiomic (quantitative) and 10 semantic (qualitative) features were applied to quantify the imaging phenotype. Area under the curve (AUC) and odd ratios (OR) were computed with multiple-hypothesis correction. Random-forest classifiers were developed and validated on an independent dataset (n = 44).

**Results:**

Twelve radiographic features (eight radiomic and four semantic) were significantly associated with meningioma grade. High-grade tumors exhibited necrosis/hemorrhage (OR_sem_ = 6.6, AUC_rad_ = 0.62–0.68), intratumoral heterogeneity (OR_sem_ = 7.9, AUC_rad_ = 0.65), non-spherical shape (AUC_rad_ = 0.61), and larger volumes (AUC_rad_ = 0.69) compared to low-grade tumors. Radiomic and sematic classifiers could significantly predict meningioma grade (AUC_sem_ = 0.76 and AUC_rad_ = 0.78). Furthermore, combining them increased the classification power (AUC_radio_ = 0.86). Clinical variables alone did not effectively predict tumor grade (AUC_clin_ = 0.65) or show complementary value with imaging data (AUC_comb_ = 0.84).

**Conclusions:**

We found a strong association between imaging features of meningioma and histopathologic grade, with ready application to clinical management. Combining qualitative and quantitative radiographic features significantly improved classification power.

## Introduction

Meningiomas are the most common primary brain tumor in adults, with most considered benign by the World Health Organization histopathologic criteria (WHO grade I)[1,2]. A distinct and increasing proportion of meningiomas are deemed high-grade (WHO grade II-III) and recur despite aggressive treatment, leading to substantial morbidity. Standard-of-care management typically involves surgical resection and often radiation therapy for high-grade (grade II-III) or progressive tumors.

Currently, the assessment of tumor grade occurs once a mass is resected and histopathological review is performed. Upon detection of a mass lesion that displays radiological features suggestive of meningioma, reliable parameters do not exist that can predict tumor grade and the associated clinical course. For example, clinical information such as age and gender show poor association with grade. Non-invasive and early predictors of meningioma grade may enhance clinical decision-making by providing prognostic information that could guide the decision of whether to observe or to treat.

**Fig 1:**
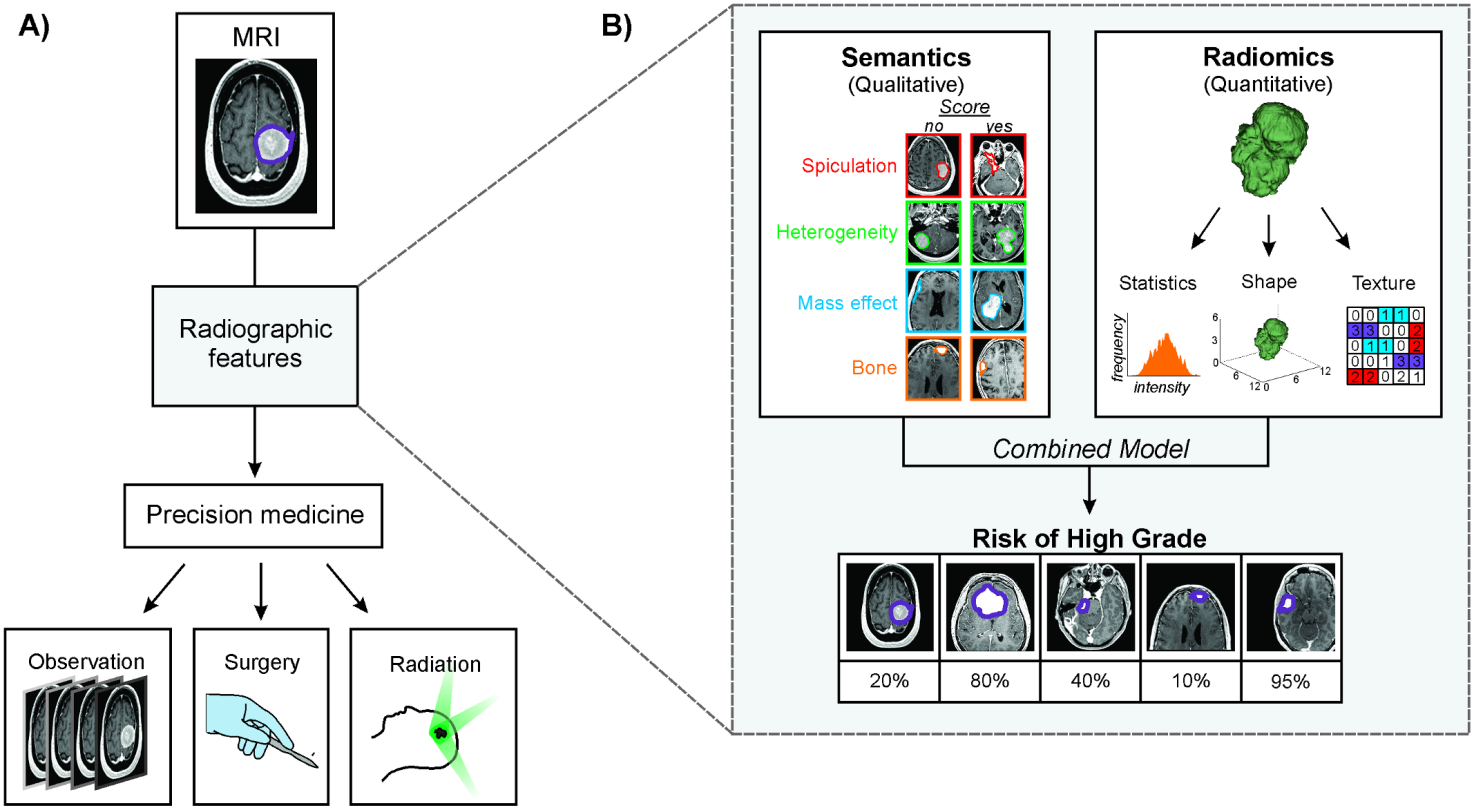
**A)** Potential impact of radiographic features on meningioma patient management. Pre-operative radiographic assessment of grade may improve the ability to tailor precision medicine decision trees to individual patients. **B)** A combined model of semantic and radiomic radiographic features was used to predict meningioma grade and validated on an independent cohort of meningiomas.

The radiographic appearance of a tumor can be described using both quantitative and qualitative measures (Fig 1). Radiomics is an emerging field of quantitative imaging focused on leveraging large sets of imaging features to create an atlas[3–9] that would foster the automatic, reproducible, and unbiased assessment of active clinical cases[10–13]. In comparison, semantic features are tumor traits (e.g. bone invasion, necrosis) that are assessed visually by radiologists. While semantic features are highly intuitive, they are inherently subject to inter-observer variability. Both radiomic[14–29] and semantic[30–33] features have been applied as prognostic biological signatures, and therefore, may offer complementary streams to predict clinical status.

In this study, we investigated the value of radiomic and semantic imaging features for predicting the histologic grade of meningiomas from preoperative gadolinium-enhanced T1-weighted MRI.

## Methods

This study was reviewed and approved by the Brigham and Women’s Hospital institutional review boards (IRB). Patient consent was waived by IRB protocol. All methods were performed in accordance with the relevant guidelines and regulations.

### Patient data

A total of 181 meningiomas resected at our institution between 2003 and 2014 were reviewed for histopathology and imaging. Pre-operative gadolinium-enhanced T1-weighted MRI sequences were chosen for analysis to represent the most frequently reviewed images for meningiomas. Six cases with motion artifact were excluded from analyses.

Histopathologic review of all tumors was performed by two board-certified neuropathologists (S.S., M.A.). Meningiomas were graded according to the 2007 World Health Organization (WHO) classification system [1]. The data were additionally reviewed according to the 2016 WHO classification system to assess any potential impact that the inclusion of brain invasion as a formal diagnostic criterion for grade II meningiomas might have on their association with imaging features. In this study, low and high grade refers to grade I and grade II/III, respectively. Atypical features for meningiomas were individually tabulated [34].

### Image-based phenotyping

In this study, semantic (qualitative) and radiomic (quantitative) feature quantification was applied to preoperative MRI (Fig 1B, Table 1). The standard preoperative imaging protocol for intracranial tumors include a high-resolution gadolinium-enhanced T1-weighted 3D MPRAGE or SPGR sequence, acquired on a 1.5T or 3T scanner. For patients who had had serial imaging prior to surgery, we analyzed the MRI that was acquired closest to the date of surgery. We exported images into 3D Slicer[35] for editing and reconstructed meningioma volumes from the manual contours of individual axial MRI slices performed by two fully trained neurosurgeons familiar with the radiographic appearance of the meningiomas to limit inclusion of radiologic artifacts. All contours were reviewed by an experienced neuroradiologist (R.H.) to ensure standardization of contouring criteria across the dataset. We applied image processing prior to feature extraction to reduce noise (mean +/- 3 standard deviations) according to well-established MRI-normalization methods. We resampled the voxel dimensions using 3x3x3 mm^3^ as the common spacing.

**Table 1:** Description of radiographic features and filters. Individual descriptions are given for each group and parameter or feature.

Type	Group	Feature / Parameter	Description
Radiographic features	Semantic	Intratumoral heterogeneity	Heterogeneity in hyperintensity of MRI signal throughout tumor
		Multifocality	Non-contiguous growth of tumor
		Midline shift	Shift of the brain past midline
		Sinus invasion	Presence of venous sinus invasion
		Necrosis / Hemorrhage	Presence of necrosis or hemorrhage
		Mass effect	Shift in normal brain parenchyma due to tumor
		Cystic component	Fluid filled cysts within the tumor
		Bone invasion	Appearance of tumor invading the skull
		Hyperostosis	Bony overgrowth adjacent to tumor
		Spiculation	Irregularities in tumor shape and border
	Radiomic	Median	Median voxel intensity value
		Mean	Mean voxel intensity value
		Minimum	Minimal voxel intensity value
		Skewness	Describes the shape of a probability distribution of the voxel intensity histogram
		Spherical Disproportion (SD)	How different is the tumor is to a sphere with a similar volume
		Cluster Prominence (CP)	Sensitive to flat zones (area of similar intensity)
		Difference Entropy (DE)	Complexity of the pattern (high entropy for high number of unique patterns)
		Inverse Difference Normalized (IDN)	Sensitive to homogeneity in the tumor
		Run Length Non-uniformity (RLN)	Measure of heterogeneity
		Short Run Low Gray-Level Emphasis (SRLGLE)	Measure of heterogeneity sensitive to low intensity pattern
		High Intensity Large Area Emphasis (HILAE)	Sensitive to flat zones with high intensity voxels (e.g. areas of hemorrhage)
		Low Intensity Large Area Emphasis (LILAE)	Sensitive to flat zones with low intensity voxels (e.g. areas of necrosis)
		Low Intensity Small Area Emphasis (LISAE)	Sensitive to small flat zones with low intensity voxels
Filters	Wavelet	High (L), Low (L)	Wavelet filters decompose images by high (increase details) and low (smooth image, leaving general shape) for every spatial component (x,y,z)
	LoG	Sigma (σ)	Laplacian of Gaussian is a filter that highlights textures using a variable size radius (σ). Depending on the radius (from 0.5mm to 5mm with 0.5 increment), it emphasizes image textures from fine to coarse.

**Fig 2:**
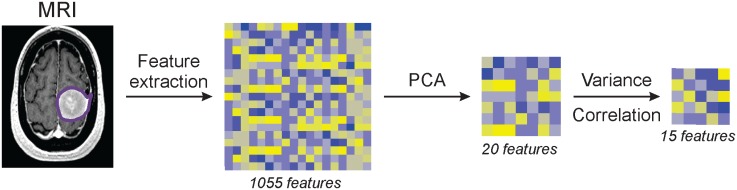
Schematic of the radiomic feature selection process from the extraction to the final feature set.

Semantic features such as speculation and mass effect are MRI characteristics regularly assessed during the standard evaluation of images from patients with meningiomas. Ten binary (to avoid high inter-observer variability) semantic features were scored by an experienced neuroradiologist (R.H.) whereas radiomic features were extracted from images using a custom Matlab script. A total of 1,055 radiomic features were computed that quantify the tumor phenotype (description in S1 File). We selected fifteen features for this study based on their variance and correlation (Fig 2).

Additionally, we included two tumor size features (maximum axial diameter and volume) in the clinical data set, along with age, gender, and radiation induced status. Tumor location was also classified by two skull base surgery trained neurosurgeons, based on the origin of the meningioma, and clustered into five groups for purposes of analysis: 1) midline skull base, 2) lateral skull base, 3) midline convexity, 4) lateral convexity or 5) other.

### Univariate analysis

All statistical analyses were performed in R software version 3.3.1[36]. Our primary endpoint was the potential applicability of radiographic features to predict meningioma grade. The predictive power of semantic features (binary) was evaluated using the odds ratio (OR) and Fisher’s exact test. The predictive power of radiomic features (continuous) was analyzed using the area under the receiver operator characteristics curve (AUC) using the “survcomp” package[37] and Noether’s test.

Additionally, prediction of low grade (grade I) with the presence of atypical features was studied. A subset of the cohort with only low grade meningioma was analyzed, where we compared patients with one or more atypical features (including spontaneous necrosis, high nuclear-to-cytoplasmic ratio, prominent nucleoli, and sheet-like growth) versus none of these features using the same imaging features as for grade prediction. Hypercellularity was almost ubiquitously observed across the meningioma cohort, and therefore, not included as an atypical feature for purposes of analysis.

Finally, the association between radiomic and semantic features was investigated using the AUC. Every semantic feature was predicted by each of the radiomic features in a univariate manner. All p-values were adjusted for multiple hypothesis testing using the false discovery rate method[38].

### Multivariate analysis

A temporal split was used to assign patients to a training or validation dataset. Feature selection was based on the training dataset, to ensure independence from the validation dataset (**Table A** in S1 File). Differences in clinical variables between datasets were assessed using the Fisher’s exact test (for categorical variables) and the Wilcoxon test (for continuous variables).

We investigated five models for grade classification based on: 1) clinical, 2) location, 3) semantic, 4) radiomic, 5) radiographic (combined radiomic and semantic features), and 6) a combined model integrating all features above. Classifications were made using the random forest method from the “randomForest” package [39]. Nested cross validation was used for model tuning and training using the “caret” package on the training set [40], leaving the validation dataset independent from the model selection process. Differences in predictive power between models were assessed using bootstrapping (1,000 iterations).

## Results

### Clinical cohort

Our cohort of 175 patients was mainly composed of female patients (62%), with a median age of 57 years (Table 2). 59% of cases were low-grade and 41% were high-grade. No differences were observed in WHO grade (p = 0.48), radiation-induced status (p = 0.51), or gender (p = 0.15) between the training and validation datasets.

**Table 2:** Demographic information across the full, training, and validation datasets.

Variable	Groups	Full (n = 175)	Training (n = 131)	Validation (n = 44)	*p-value*
Age (years)	Median (range)	57 (22–89)	57 (22–89)	57.5 (29–89)	***0*.*28***
Gender	Male	68 (38%)	55 (42%)	13 (30%)	***0*.*15***
	Female	107 (62%)	76 (58%)	31 (70%)	
WHO grade	Low (grade I)	103 (59%)	75 (57%)	28 (63%)	***0*.*48***
	High (grade II-III)*	72 (41%)	56 (43%)	16 (37%)	
	Grade II	66	52	14	
	Grade III	6	4	2	
Grade 1 meningioma with atypical features	No	69	49	20	***0*.*68***
	Yes	34	26	8	
Radiation-induced	Yes	13 (7%)	11 (8.3%)	2 (4.5%)	***0*.*51***
	No	160 (93%)	120 (91.7%)	42 (95.5%)	
Location	Midline Skull-base	23	17	6	***0*.*70***
	Lateral Skull-base	53	41	12	
	Midline Convexity	39	31	8	
	Lateral Convexity	53	37	16	
	Other	5	3	5	

* including 3 chordoid (grade II) and 1 rhaboid (grade III)

### Radiographic associations with meningioma grade or atypical features

**Fig 3:**
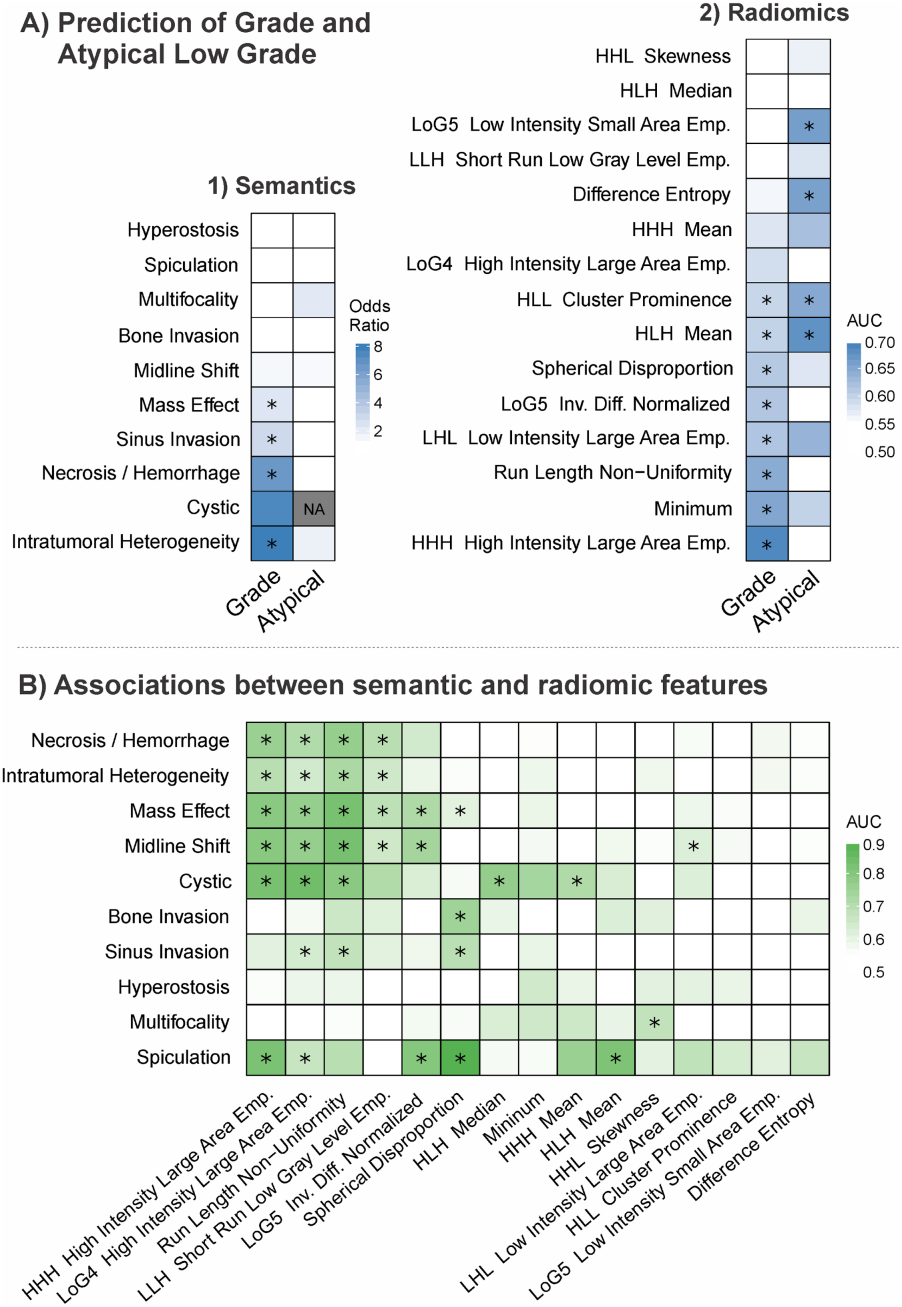
**A)** Heatmap of the predictive power of (1) semantic and (2) radiomic features for meningioma grade (n = 175) or presence of histopathologic atypia in low grade meningiomas (n = 103). **B)** The association between semantic and radiomic features was investigated. Every semantic feature was predicted with each of the radiomic feature in a univariate manner that indicates their relationship. * indicates significance from random after multiple correction.

First, examination of individual semantic (qualitative) features revealed significant associations between meningioma grade and four features (Fig 3A.1, Table 3). These features included intratumoral heterogeneity (OR = 7.9, p<0.001), necrosis/hemorrhage (OR = 6.6, p = 0.01), venous sinus invasion (OR = 2.9, p = 0.02), and mass effect (OR = 2.3, p = 0.042). Interestingly, cystic component was not significantly associated with grade despite a high OR (6.8, p = 0.13), which is likely due a low incidence of events (6 cases, 3.4%), which introduces a high margin error. All significant features had an OR greater than one, indicating that higher grade corresponds to an increased incidence of the feature.

**Table 3:** Univariate results for the semantic features. Odds ratio, lower and higher 95% confidence interval and p-value (with multiple testing correction) are reported for each features.

	Odds Ratio	95% Conf. Int.		p-value
Hyperostosis	0.35	0.08	1.16	0.14
Spiculation	0.47	0.01	6.01	0.81
Multifocality	0.89	0.22	3.23	1.00
Bone Invasion	1.00	0.31	3.09	1.00
Midline Shift	1.39	0.71	2.70	0.49
Mass Effect	2.31	1.20	4.53	0.02
Sinus Invasion	2.91	1.33	6.59	0.02
Necrosis / Hemorrhage	6.60	1.69	37.88	0.01
Cystic	7.53	0.82	362.79	0.14
Intratumoral Heterogeneity	7.95	3.62	18.83	<0.001

Second, we investigated the relationship between radiomic (quantitative) features and meningioma pathology (Fig 3A.2, Table 4). Eight radiomic features were significant from random in their association with tumor grade (range AUC = 0.59 to 0.65, p<0.05). The best performing radiomic feature, high intensity large area emphasis (HILAE), was associated with high grade meningioma (AUC = 0.69, p<0.001). HILAE is sensitive to large zones with high intensities (e.g. hemorrhage). In addition, low intensity large area emphasis (LILAE) was also associated with high grade meningioma (AUC = 0.63, p = 0.008) and is sensitive to large areas of low intensities (e.g. necrosis). These suggest that hemorrhagic or necrotic tumors were more likely to be high grade, consistent with the semantic feature analysis. High values of spherical disproportion (SD), which measures the degree of deviation of a tumor’s shape from a sphere of similar volume, and run length non-uniformity (RLN), which is sensitive to heterogeneity, were both significantly associated with high-grade tumors (AUC = 0.61, p = 0.012 and AUC = 0.65, p = 0.002, respectively).

**Table 4:** Univariate results for the radiomic features. AUC, lower and higher 95% confidence interval and p-value (with multiple testing correction) are reported for each features.

Features	AUC	95% Conf. Int.		p-value
HHL Skewness	0.51	0.40	0.57	0.74
HLH Median	0.52	0.44	0.61	0.64
LoG5 Low Intensity Small Area Emp.	0.54	0.46	0.63	0.35
LLH Short Run Low Gray Level Emp.	0.55	0.37	0.54	0.35
Difference Entropy	0.56	0.35	0.52	0.21
HHH Mean	0.58	0.50	0.66	0.09
LoG4 High Intensity Large Area Emp.	0.59	0.50	0.67	0.08
HLL Cluster Prominence	0.60	0.51	0.68	0.05
Spherical disproportion	0.61	0.53	0.69	0.02
LoG5 Inv. Diff. Normalized	0.61	0.53	0.70	0.02
HLH Low Intensity Large Area Emp.	0.63	0.54	0.71	0.01
HHL Mean	0.63	0.55	0.71	<0.001
Run Length Non-uniformity	0.65	0.56	0.73	<0.001
Minimum	0.65	0.57	0.73	<0.001
HHH High Intensity Large Area Emp.	0.69	0.61	0.77	<0.001

Additionally, we examined the ability of imaging to distinguish low grade meningiomas with (n = 69) and without (n = 34) one of four atypical features (Fig 3A, **Tables B-C** in S2 File). While intratumoral heterogeneity and multifocality carried an OR of 1.7 and 2.1, respectively, no significant association was observed between semantic features and the presence of atypical features. In comparison, four radiomic features were significantly associated with atypical features. These features included voxel mean intensity (AUC = 0.68), low intensity small area emphasis (LISAE) (AUC = 0.66), difference entropy (DE) (AUC = 0.66), and cluster prominence (CP) (AUC = 0.65). LISAE indicated that hypointense tumors were more likely to be low grade with atypical features. None of the low grade meningiomas had a cystic component; therefore, this semantic feature was not investigated in this analysis.

### Relationship between radiomic and semantic features

We investigated the link between radiomic and semantic features. We found a median AUC of 0.57 (range: 0.50–0.89) between these two categories of features (Fig 3B, **Table D** in S2 File). A significant interaction between particular pairs of features was found (p<0.05). Spherical disproportion (SD) was associated with mass effect (AUC = 0.61), spiculation (AUC = 0.89), and invasion of bone and venous sinus (AUC of 0.74 and 0.69, respectively). Textural features, such as high intensity large area emphasis (HILAE), run length non-uniformity (RLN) and short run length gray-level (SRLGL) were associated with tumor heterogeneity (AUC = 0.65–0.72), cystic component (AUC = 0.71–0.84), and hemorrhage / necrosis (AUC = 0.70–0.76).

### Improving grade classification by combining radiographic features

**Fig 4:**
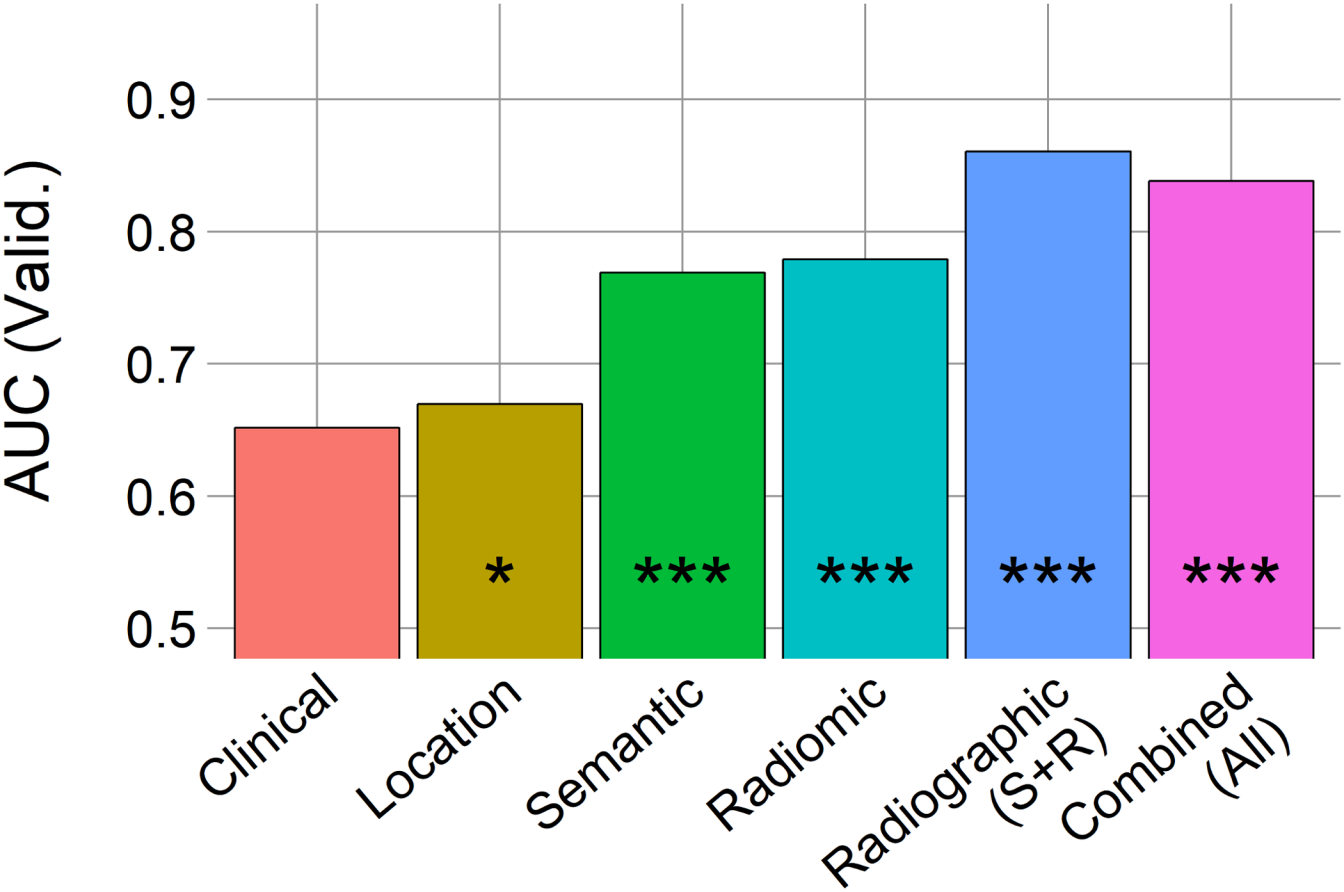
Area under the curve (AUC) from random forest models on the independent validation set (n = 44) for meningioma grade classification. “*” indicates p-value <0.05, “***” indicates p-value <0.0001 from random prediction (Noether test).

Given that radiomic and semantic analyses each provide a distinct quantification of the tumor phenotype, we explored whether combining radiomic and semantic features may be synergistic in predicting meningioma grade (Fig 4, Table 5).

**Table 5:** Meningioma classification validation (n = 44) for each model is reported. AUC, lower and higher 95% confidence interval and p-value (from random) are reported for each features.

	AUC	Sensitivity	Specificity	95% Conf. Int.		p-values
Clinical	0.651786	0.928571	0.375	0.468065	0.835507	0.105389
Location	0.669643	0.892857	0.3125	0.541902	0.907802	0.016002
Semantic	0.768973	0.75	0.625	0.648494	0.913278	3.21E-05
Radiomic	0.779018	0.928571	0.625	0.639093	0.918943	9.30E-05
Radiographic (Loc. + Sem. + Rad.)	0.860491	0.821429	0.625	0.762583	0.960012	7.31E-13
Combined (All)	0.83817	0.892857	0.375	0.733099	0.944753	3.45E-10

A model based on clinical data, composed of information available to a clinician prior to MR imaging, did not associate with meningioma grade (AUC = 0.65, p = 0.11). In comparison, location (AUC = 0.67), semantic (AUC = 0.77) and radiomic (AUC = 0.79) models independently classified meningioma grade (p<0.05). Interestingly, a radiographic model that combined both radiomic and semantic features showed an increased performance in the classification of tumor grade (AUC = 0.86, p<0.001). Despite the fact that the performance of the radiographic model was higher than the semantic and radiomic models, it was not significantly better than each alone (p-value = 0.23–0.32). Lastly, adding clinical and location data to the radiographic model did not improve the performance (AUC = 0.84) compared to radiographic features (AUC = 0.86).

Additionally, we verified the validity of the imaging association with meningioma grade using the 2016 WHO guidelines, which includes brain invasion as a formal inclusion criterion for grade II. We observed similar results with the updated WHO criteria as the 2007 criteria, with additional observation of a significant association between the clinical model and pathologic grade (in **Fig A, Table E** in S2 File), attesting to the robustness of the radiographic association with tumor grade.

## Discussion

Meningioma grade is a powerful predictor of clinical outcome and therefore influences patient management, including the decision of whether to observe, operate, or administer adjuvant therapies. Currently, tumor grade can only be determined following surgical resection and histopathological review[41]. A better approach would allow clinicians to discriminate low and high grade meningiomas before surgery, thereby facilitating management decisions and counseling at an earlier stage of clinical care. Such a shift in the diagnostic paradigm would have substantial implications for patient management, particularly in the increasingly common scenario era in which asymptomatic meningiomas are incidentally diagnosed on imaging performed for unrelated reasons. In our study, we sought to develop and test methodologies for the pre-operative diagnostic assessment of meningioma grade using two categories of radiographic data (semantic and radiomic) derived from T1-weighted contrast-enhanced MRI.

We observed strong associations between specific radiographic features and meningioma histologic grade. In particular, heterogeneous tumors with necrosis and/or hemorrhage, and irregularly shaped (non-spherical) tumors were more likely to be higher grade on univariate analysis. Two radiomic features, HILAE and LILAE, were sensitive to high and low intensity large areas, respectively, which are commonly indicative of hemorrhage and necrosis on MR images. Interestingly, both semantic and radiomic features were significantly associated with these traits and their presence indicated an increased likelihood of a high grade tumor. Tumor heterogeneity was also significantly associated with more aggressive meningioma grade in both semantic and radiomic feature analyses.

Irregularities in the shape of meningiomas such as “mushrooming” has been previously associated with high grade tumor in multiple studies[42–45]. Meningioma heterogeneity, on the other hand, is a more complex tumor trait that may be accounted for by a variety of underlying causes, including intratumoral necrosis, cystic degeneration, heterogeneous tumor cell expansion, variability in cell density, and hemorrhage[46,47]. Tumor radiographic heterogeneity has been extensively studied in glioblastoma, lung cancer, renal cell cancer, and other systemic malignancies and is felt to contribute significantly to treatment resistance and disease relapse[48]. Awareness of tumor heterogeneity may play an important role in assessing treatment response in meningiomas as well, given recent and impending clinical trials assessing novel targeted and immune therapies for aggressive meningiomas[49–51].

We further confirmed the ability of radiographic features to classify meningioma grade on an independent validation dataset. Moreover, even though semantic and radiomic features capture some common traits in the tumor (e.g. heterogeneity), the information contained in these “common” features were complementary. Merging both feature sets significantly improved classification performance, indicating an additive effect between qualitative and quantitative imaging analyses. Additionally, tumor location alone was able to classify tumor grade but was not able to improve the combined model once added to the radiographic and clinical information (likely mostly driven by the radiographic features). Clinical data, added for comparison, did not classify patients well nor did it add power to the radiographic model in the validation, although this could be limited by selection bias in the variables analyzed.

Associations between tumor characteristics and pre-operative images have been previously investigated[52]. However, no sets of phenotypic features have been consistently demonstrated to significantly associate with meningioma grade across studies. Differences between meningioma and low grade glioma was investigated using imaging features from T1-weighed and DWI[53], however, the study presented several limitations including a small sample size (n = 15). Some studies investigating imaging features suggest that benign tumors display higher ADC while malignant tumors have lower ADC values[54–56], while others fail to corroborate a similar relationship [57–59]. These conflicting results may be due to technical factors, such as the region of interest (ROI) defined and feature standardization[59,60].

Likewise, our study faces several limitations. Variations in image acquisition and quality can influence quantitative analyses. We attempted to standardize the uniformity of scans by resampling all images with a common voxel spacing to ensure dimension homogeneity and by filtering voxel intensities to reduce outlier values. Additionally, the semantic features are reported by human thus are subject to inter-observer variability. To reduce this variation, we used largely binary features to simply the output, as compared to a more complex scale (such as a score from 1 to 5) which have been suggested to associate with more inter-observer variability [61]. We used a temporal split to obtain an independent validation dataset, with comparable demographics between the cohorts, in attempt to internally validate our results. External validation from multiple institutions would strengthen these observations in the future. Our clinical model was predicated on common non-radiographic variables that may influence tumor behavior, but may reflect selection bias and data availability in this single-institution cohort.

In conclusion, we found a radiographic signature for meningioma grade using standard pre-operative contrast-enhanced MR images. We demonstrated that there is a strong link between the radiographic phenotype of a tumor and its pathology, which may provide a useful tool for precision medicine. Early and accurate prediction of meningioma grade may influence the decision to observe a tumor or to pursue surgery and earlier consideration of adjuvant therapies. Our study highlights the potential clinical impact of integrative imaging analysis in guiding meningioma management.

## Supporting information

S1 File Table A. Description of the training radiomic set. Individual description is given for every features.
(DOCX)

S2 File **Table B** Univariate results for the semantic features. Odds ratio, lower and higher 95% confidence interval and p-value (with multiple testing correction) are reported for each features. **Table C** Univariate results for the radiomic features. AUC, lower and higher 95% confidence interval and p-value (with multiple testing correction) are reported for each features. **Table D.** Association between radiomic and semantic features was investigated using AUC. **Table E.** Meningioma classification validation (n = 44) for each model is reported using the WHO 2016. AUC, lower and higher 95% confidence interval and p-value (from random) are reported for each features. **Fig A.** Area under the curve (AUC) from random forest models on the independent validation set (n = 44) for meningioma WHO 2016 grade classification. “*” indicates p-value <0.05, “***” indicates p-value <0.0001 from random prediction (Noether test).
(DOCX)
