# Supplementary material for: Radiographic prediction of meningioma grade by semantic and radiomic features
Source: PLoS One. 2017 Nov 16;12(11):e0187908. doi: 10.1371/journal.pone.0187908 (PMC5690632; doi:10.1371/journal.pone.0187908)
Supplement: S1 File — Description of the training radiomic set. Individual description is given for every features. (DOCX) [file pone.0187908.s001.docx]

**S1:** Radiomic feature selection

A total of 1055 radiomic features [1] that quantify phenotype were extracted for this analysis from the primary tumor. Individual descriptions of the features and filters can be found in the supplement material from a previous study [2]. Using data mining techniques based on feature variance and correlation, we selected fifteen features for this study. Only these fifteen features were included in our analysis to evaluate their power to predict meningioma tumor grade (low/high). Dimension reduction was performed in R software [3] version 3.3.1.

**Principal Component Analysis**

From those 1055 features, we used Principal Component Analysis (PCA) and factor analysis to reduce the resulting high-dimensional dataset to a low-dimensional dataset while retaining most of the variation contained within the data. PCA creates a new principal component space using value decomposition of the features, also called scores.

We selected scores retaining 95% of the variability from the primary tumor; we then selected features that correlated by at least 99% to the PCA scores. PCA analysis was performed using the ”factoMineR” package [4]. After this procedure, 20 features from the primary tumor were obtained based on user-set parameters.

**Correlation / Variance selection**

After PCA selection, we excluded features based on correlation. Any features that had more than absolute 0.7 correlation were excluded (the features with the highest overall correlation was excluded). Finally, we kept the 15 features with the highest variance.

**Multivariate analysis**

For multivariate purpose (and avoid leak of information between training and validation), the features were reselected on the training only using the same methodology. The newly selected features are shown in the **Table A.** The semantic set is unchanged as no selection was applied on it.

| Type | Group | features | Description |
| --- | --- | --- | --- |
| Radiographic features | Radiomic | Mean | Mean voxel intensity value |
|  |  | Minimum | Min voxel intensity value |
|  |  | Kurtosis | Describe the shape of a probability distribution of the voxel intensity histogram |
|  |  | Skewness | Describe the shape of a probability distribution of the voxel intensity histogram |
|  |  | Long Run Emphasis (LRE) | Associated with pattern involving long stripes |
|  |  | Large Area Emphasis (LAE) | Sensitive to large flat zones (areas of connecting voxels with the same value) |
|  |  | Correlation | Correlation of the patterns (smooth gradients in the patterns) |
|  |  | Inverse Difference Normalized (IDN) | Sensitive to homogeneity in the tumor |
|  |  | Run Length Non-uniformity (RLN) | Measure of heterogeneity |
|  |  | High Intensity Large Area Emphasis (HILAE) | Sensitive to flat zones with high intensity voxels (e.g. areas of hemorrhage) |
|  |  | Low Intensity Large Area Emphasis (LILAE) | Sensitive to flat zones with low intensity voxels (e.g. areas of necrosis) |

**Table A.** Description of the training radiomic set. Individual description is given for every features.

**References**

1. Lambin P, Rios-Velazquez E, Leijenaar R, et al (2012) Radiomics: Extracting more information from medical images using advanced feature analysis. Eur J Cancer 48:441–446. doi: 10.1016/j.ejca.2011.11.036

2. Coroller TP, Grossmann P, Hou Y, et al (2015) CT-based radiomic signature predicts distant metastasis in lung adenocarcinoma. Radiother Oncol 114:345–350. doi: 10.1016/j.radonc.2015.02.015

3. R Core Team (2013). R: A language and environment for statistical computing. R Foundation for Statistical Computing, Vienna, Austria. ISBN 3-900051-07-0, URL http://www.R-project.org/.

4. Husson F, Josse J, Le S, Mazet J (2015) FactoMineR: Multivariate Exploratory Data Analysis and Data Mining.
