# Supplementary material for: Radiographic prediction of meningioma grade by semantic and radiomic features
Source: PLoS One. 2017 Nov 16;12(11):e0187908. doi: 10.1371/journal.pone.0187908 (PMC5690632; doi:10.1371/journal.pone.0187908)
Supplement: S2 File — Table B Univariate results for the semantic features. Odds ratio, lower and higher 95% confidence interval and p-value (with multiple testing correction) are reported for each features. Table C Univariate results for the radiomic features. AUC, lower and higher 95% confidence interval and p-value (with multiple testing correction) are reported for each features. Table D. Association between radiomic and semantic features was investigated using AUC. Table E. Meningioma classification validation (n = 44) for each model is reported using the WHO 2016. AUC, lower and higher 95% confidence interval and p-value (from random) are reported for each features. Fig A. Area under the curve (AUC) from random forest models on the independent validation set (n = 44) for meningioma WHO 2016 grade classification. “*” indicates p-value <0.05, “***” indicates p-value <0.0001 from random prediction (Noether test). (DOCX) [file pone.0187908.s002.docx]

**S2:** Results details

**Atypical features grade I prediction**

|  | Odds Ratio | 95% Conf. Int. | | p-value |
| --- | --- | --- | --- | --- |
| Spiculation | 0.00 | 0.00 | 4.92 | 0.84 |
| Sinus Invasion | 0.46 | 0.08 | 1.90 | 0.84 |
| Bone Invasion | 0.48 | 0.05 | 2.61 | 0.84 |
| Hyperostosis | 0.71 | 0.15 | 2.65 | 0.85 |
| Mass Effect | 0.81 | 0.32 | 2.01 | 0.84 |
| Necrosis / Hemorrhage | 1.02 | 0.02 | 20.16 | 1.00 |
| Midline Shift | 1.32 | 0.51 | 3.37 | 0.84 |
| Intratumoral Heterogeneity | 1.75 | 0.70 | 4.39 | 0.84 |
| Multifocality | 2.15 | 0.37 | 12.38 | 0.84 |
| Cystic | - | - | - | - |

**Table B** Univariate results for the semantic features. Odds ratio, lower and higher 95% confidence interval and p-value (with multiple testing correction) are reported for each features.

| Features | AUC | 95% Conf. Int. | | p-value |
| --- | --- | --- | --- | --- |
| LoG5 Inv. Diff. Normalized | 0.50 | 0.38 | 0.62 | 0.96 |
| HLH Median | 0.50 | 0.39 | 0.62 | 0.96 |
| Run Length Non-Uniformity | 0.51 | 0.37 | 0.60 | 0.93 |
| LoG4 High Intensity Large Area Emp. | 0.53 | 0.35 | 0.59 | 0.74 |
| HHH High Intensity Large Area Emp. | 0.54 | 0.42 | 0.66 | 0.74 |
| HHL Skewness | 0.57 | 0.44 | 0.69 | 0.43 |
| Spherical Disproportion | 0.58 | 0.31 | 0.53 | 0.30 |
| LLH Short Run Low Gray Level Emp. | 0.58 | 0.45 | 0.70 | 0.36 |
| Minimum | 0.60 | 0.51 | 0.71 | 0.17 |
| HHH Mean | 0.62 | 0.51 | 0.73 | 0.08 |
| LHL Low Intensity Large Area Emp. | 0.64 | 0.52 | 0.75 | 0.06 |
| HLL Cluster Prominence | 0.65 | 0. 53 | 0.76 | 0.05 |
| Difference Entropy | 0.66 | 0.54 | 0.77 | 0.04 |
| LoG5 Low Intensity Small Area Emp. | 0.66 | 0.54 | 0.78 | 0.04 |
| HLH Mean | 0.68 | 0.57 | 0.78 | 0.01 |

**Table C** Univariate results for the radiomic features. AUC, lower and higher 95% confidence interval and p-value (with multiple testing correction) are reported for each features.

**Association between semantic and radiomic features**

Relationship between radiomic and semantic features was investigated prior clinical outcomes prediction. Due to variable nature (radiomic = continuous, sematic = binary variable), AUC was used as surrogated for their association (**Table D**).

|  | HHH High Intensity Large Area Emp. | LoG4 High Intensity Large Area Emp. | Run Length Non-Uniformity | LLH Short Run Low Gray Level Emp. | LoG5 Inv. Diff. Normalized | Spherical Disproportion | HLH Median | Minimum | HHH Mean | HLH Mean | HHL Skewness | LHL Low Intensity Large Area Emp. | HLL Cluster Prominence | LoG5 Low Intensity Small Area Emp. | Difference Entropy |
| --- | --- | --- | --- | --- | --- | --- | --- | --- | --- | --- | --- | --- | --- | --- | --- |
| Necrosis / Hemorrhage | 0.76 | 0.71 | 0.77 | 0.70 | 0.65 | 0.53 | 0.52 | 0.56 | 0.51 | 0.54 | 0.52 | 0.56 | 0.51 | 0.58 | 0.56 |
| Intratumoral Heterogeneity | 0.70 | 0.65 | 0.72 | 0.65 | 0.59 | 0.56 | 0.51 | 0.59 | 0.55 | 0.53 | 0.58 | 0.52 | 0.51 | 0.58 | 0.56 |
| Mass Effect | 0.79 | 0.77 | 0.83 | 0.69 | 0.72 | 0.61 | 0.55 | 0.59 | 0.54 | 0.51 | 0.51 | 0.59 | 0.56 | 0.54 | 0.52 |
| Midline Shift | 0.80 | 0.77 | 0.83 | 0.65 | 0.74 | 0.52 | 0.51 | 0.57 | 0.55 | 0.58 | 0.56 | 0.62 | 0.57 | 0.51 | 0.56 |
| Cystic | 0.82 | 0.84 | 0.79 | 0.71 | 0.63 | 0.57 | 0.77 | 0.73 | 0.71 | 0.63 | 0.52 | 0.62 | 0.51 | 0.54 | 0.53 |
| Bone Invasion | 0.54 | 0.57 | 0.66 | 0.62 | 0.52 | 0.75 | 0.59 | 0.55 | 0.52 | 0.63 | 0.62 | 0.53 | 0.51 | 0.54 | 0.59 |
| Sinus Invasion | 0.61 | 0.64 | 0.68 | 0.61 | 0.58 | 0.69 | 0.54 | 0.60 | 0.55 | 0.50 | 0.52 | 0.52 | 0.53 | 0.54 | 0.52 |
| Hyperostosis | 0.56 | 0.59 | 0.59 | 0.53 | 0.52 | 0.54 | 0.53 | 0.65 | 0.59 | 0.55 | 0.61 | 0.61 | 0.59 | 0.55 | 0.54 |
| Multifocality | 0.51 | 0.51 | 0.56 | 0.54 | 0.57 | 0.56 | 0.63 | 0.65 | 0.65 | 0.60 | 0.68 | 0.55 | 0.55 | 0.53 | 0.55 |
| Spiculation | 0.82 | 0.67 | 0.70 | 0.52 | 0.80 | 0.89 | 0.57 | 0.57 | 0.76 | 0.81 | 0.61 | 0.68 | 0.64 | 0.61 | 0.67 |

**Table D.** Association between radiomic and semantic features was investigated using AUC.

**WHO 2016 Grade prediction**

Using WHO 2016 grading system, we replicated the multivariate analysis (**Fig A**). Using the new grades, we found consistent results at the difference that the clinical information was now associated to the grade information. Thus, the combined model reached the highest AUC in the validation.

**
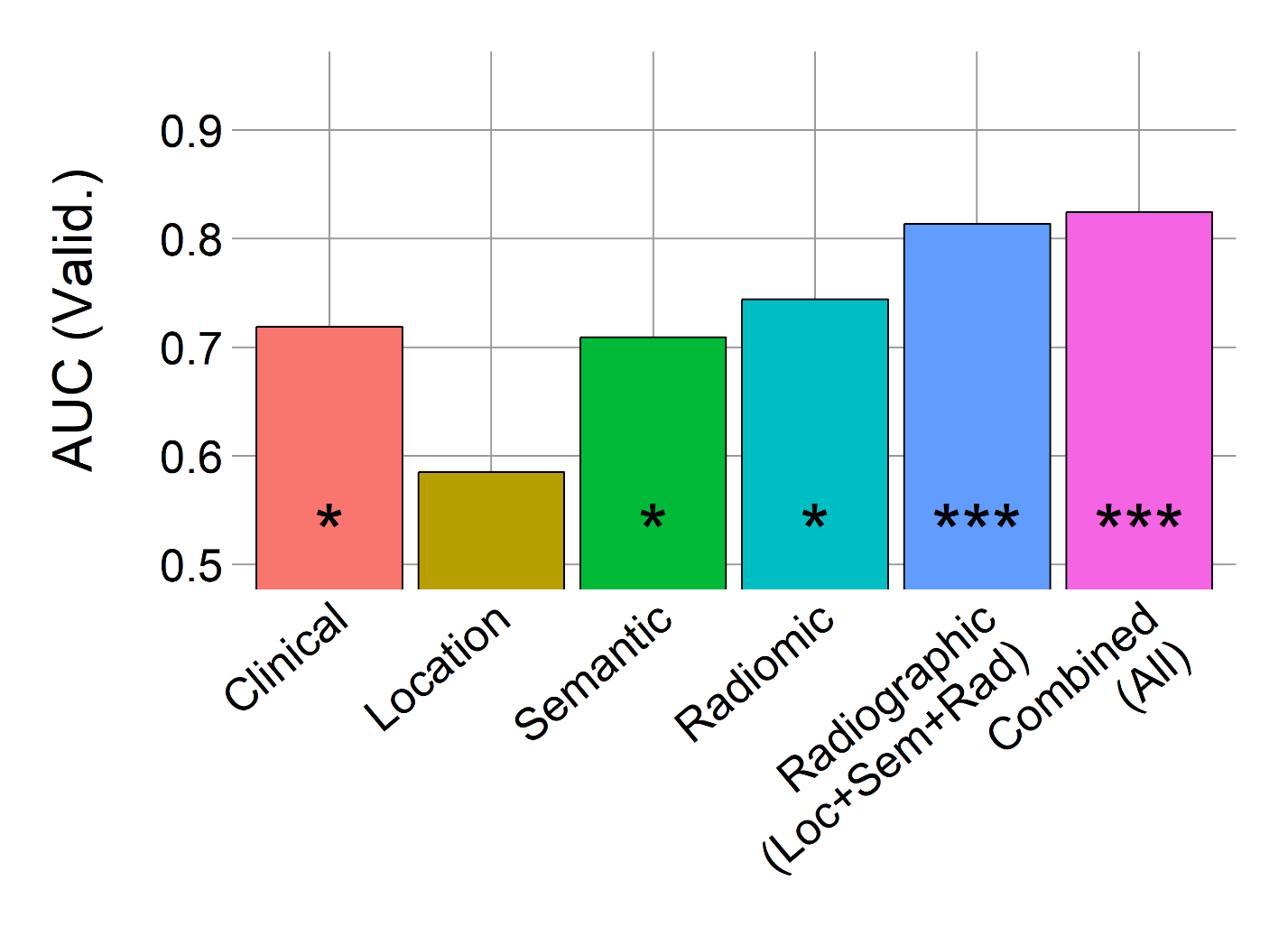
**

**Fig A.** Area under the curve (AUC) from random forest models on the independent validation set (n=44) for meningioma WHO 2016 grade classification. “*” indicates p-value <0.05, “***” indicates p-value <0.0001 from random prediction (Noether test).

|  | AUC | Sensitivity | Specificity | lower | upper | p.values |
| --- | --- | --- | --- | --- | --- | --- |
| Clinical | 0.718954 | 0.888889 | 0.411765 | 0.574587 | 0.865238 | 0.003018 |
| Location | 0.584967 | 0.888889 | 0.294118 | 0.390842 | 0.835902 | 0.318018 |
| Semantic | 0.70915 | 0.740741 | 0.588235 | 0.574048 | 0.862316 | 0.003008 |
| Radiomic | 0.744009 | 0.814815 | 0.647059 | 0.586321 | 0.902762 | 0.002451 |
| Combined | 0.824619 | 0.925926 | 0.470588 | 0.710372 | 0.943137 | 3.74E-08 |
| Radiographic | 0.813725 | 0.851852 | 0.411765 | 0.700529 | 0.928292 | 6.26E-08 |

**Table E.** Meningioma classification validation (n=44) for each model is reported using the WHO 2016. AUC, lower and higher 95% confidence interval and p-value (from random) are reported for each features.
